# Seroprevalence of SARS-CoV-2 Among Frontline Health Care Personnel in a Multistate Hospital Network — 13 Academic Medical Centers, April–June 2020

**DOI:** 10.15585/mmwr.mm6935e2

**Published:** 2020-09-04

**Authors:** Wesley H. Self, Mark W. Tenforde, William B. Stubblefield, Leora R. Feldstein, Jay S. Steingrub, Nathan I. Shapiro, Adit A. Ginde, Matthew E. Prekker, Samuel M. Brown, Ithan D. Peltan, Michelle N. Gong, Michael S. Aboodi, Akram Khan, Matthew C. Exline, D. Clark Files, Kevin W. Gibbs, Christopher J. Lindsell, Todd. W. Rice, Ian D. Jones, Natasha Halasa, H. Keipp Talbot, Carlos G. Grijalva, Jonathan D. Casey, David N. Hager, Nida Qadir, Daniel J. Henning, Melissa M. Coughlin, Jarad Schiffer, Vera Semenova, Han Li, Natalie J. Thornburg, Manish M. Patel, CDC COVID-19 Response Team, IVY Network, IVY Network, Adrienne Baughman, Kimberly W. Hart, Robert McClellan, Rendie McHenry, Jakea Johnson, Andrea Fletcher, Curtis Rich, Kemberlyne Cordero, Lori Kozikowski, Lesley De Souza, Sarah Romain, Scott Ouellette, Andres Santana, Sherell Thornton-Thompson, Michelle Howell, Jennifer Peers, Shelby Shelton, Lani Finck, Kirsten Soules, Michael Klausner, Ximena Calderon-Morales, Heidi L. Erickson, Audrey Hendrickson, Jamie Stang, Ellen Maruggi, Alex Dunn, Eddie Stenehjem, Intermountain Healthcare, Valerie Aston, Mikaele Bown, Michelle Matheu, Rilee Smith, Olivia Krol, Andrew Salar, Oregon Health, Makrina Kamel, Oregon Health, Kelly Nguyen, Peter Huynh, Sarah Karow, Michelle Bright, Holly Bookless, Sandy Mullins, Kelly Neidert, Dina McGowan, Elizabeth Cassandra, Emily Brown, Claire Carlin, Trina Wemlinger, Breona Edwards, Lori Flores, Mary LaRose, Kathie J. Ferbas, Rachel Martin-Blais, Grace M. Aldrovandi, Olivia Thompson, Sakshi Sehgal, CDC COVID-19 Response Team, Mohammed Ata Ur Rasheed, Lisa Mills, Sandra N. Lester, Brandi Freeman, Bailey Alston, Muyiwa Ategbole, Peter Browning, Li Cronin, Ebenezer David, Rita Desai, Monica Epperson, Yamini Gorantla, Tao Jia, Pete Maniatis, Kristina Ortiz, So Hee Park, Palak Patel, Yunlong Qin, Heather Tatum, Briana Zellner

**Affiliations:** ^1^Vanderbilt University Medical Center, Nashville, Tennessee; ^2^CDC COVID-19 Response Team; ^3^Baystate Medical Center, Springfield, Massachusetts; ^4^Beth Israel Deaconess Medical Center, Boston, Massachusetts; ^5^University of Colorado School of Medicine, Aurora, Colorado; ^6^Hennepin County Medical Center, Minneapolis, Minnesota; ^7^Intermountain Healthcare, Salt Lake City, Utah; ^8^Montefiore Medical Center, Bronx, New York; ^9^Oregon Health & Sciences University Hospital, Portland, Oregon; ^10^Ohio State University Wexner Medical Center, Columbus, Ohio; ^11^Wake Forest University Baptist Medical Center, Winston-Salem, North Carolina; ^12^Johns Hopkins Hospital, Baltimore, Maryland; ^13^UCLA Medical Center, Los Angeles, California; ^14^Harborview Medical Center, Seattle, Washington.; Vanderbilt University Medical Center, Nashville, Tennessee; Vanderbilt University Medical Center, Nashville, Tennessee; Vanderbilt University Medical Center, Nashville, Tennessee; Vanderbilt University Medical Center, Nashville, Tennessee; Vanderbilt University Medical Center, Nashville, Tennessee; Vanderbilt University Medical Center, Nashville, Tennessee; Vanderbilt University Medical Center, Nashville, Tennessee; Vanderbilt University Medical Center, Nashville, Tennessee; Baystate Medical Center, Springfield, Massachusetts; Baystate Medical Center, Springfield, Massachusetts; Baystate Medical Center, Springfield, Massachusetts; Baystate Medical Center, Springfield, Massachusetts; Baystate Medical Center, Springfield, Massachusetts; Baystate Medical Center, Springfield, Massachusetts; University of Colorado School of Medicine, Aurora, Colorado; University of Colorado School of Medicine, Aurora, Colorado; University of Colorado School of Medicine, Aurora, Colorado; University of Colorado School of Medicine, Aurora, Colorado; University of Colorado School of Medicine, Aurora, Colorado; University of Colorado School of Medicine, Aurora, Colorado; University of Colorado School of Medicine, Aurora, Colorado; Hennepin County Medical Center, Minneapolis, Minnesota; Hennepin County Medical Center, Minneapolis, Minnesota; Hennepin County Medical Center, Minneapolis, Minnesota; Hennepin County Medical Center, Minneapolis, Minnesota; Hennepin County Medical Center, Minneapolis, Minnesota; Salt Lake City, Utah; Intermountain Healthcare, Salt Lake City, Utah; Intermountain Healthcare, Salt Lake City, Utah; Intermountain Healthcare, Salt Lake City, Utah; Intermountain Healthcare, Salt Lake City, Utah; Oregon Health & Sciences University Hospital, Portland, Oregon; Sciences University Hospital, Portland, Oregon; Sciences University Hospital, Portland, Oregon; Oregon Health & Sciences University Hospital, Portland, Oregon; Oregon Health & Sciences University Hospital, Portland, Oregon; Ohio State University Wexner Medical Center, Columbus, Ohio; Ohio State University Wexner Medical Center, Columbus, Ohio; Ohio State University Wexner Medical Center, Columbus, Ohio; Ohio State University Wexner Medical Center, Columbus, Ohio; Ohio State University Wexner Medical Center, Columbus, Ohio; Ohio State University Wexner Medical Center, Columbus, Ohio; Ohio State University Wexner Medical Center, Columbus, Ohio; Ohio State University Wexner Medical Center, Columbus, Ohio; Ohio State University Wexner Medical Center, Columbus, Ohio; Ohio State University Wexner Medical Center, Columbus, Ohio; Ohio State University Wexner Medical Center, Columbus, Ohio; Wake Forest University Baptist Medical Center, Winston-Salem, North Carolina; Wake Forest University Baptist Medical Center, Winston-Salem, North Carolina; UCLA Medical Center, Los Angeles, California; UCLA Medical Center, Los Angeles, California; UCLA Medical Center, Los Angeles, California; Harborview Medical Center, Seattle, Washington; Harborview Medical Center, Seattle, Washington; CDC COVID-19 Response Team; CDC COVID-19 Response Team; CDC COVID-19 Response Team; CDC COVID-19 Response Team; CDC COVID-19 Response Team; CDC COVID-19 Response Team; CDC COVID-19 Response Team; CDC COVID-19; Response Team; CDC COVID-19 Response Team; CDC COVID-19 Response Team; CDC COVID-19 Response Team; CDC COVID-19 Response Team; CDC COVID-19 Response Team; CDC COVID-19 Response Team; CDC COVID-19 Response Team; CDC COVID-19; Response Team; CDC COVID-19 Response Team; CDC COVID-19 Response Team; CDC COVID-19 Response Team; CDC COVID-19 Response Team.

Health care personnel (HCP) caring for patients with coronavirus disease 2019 (COVID-19) might be at high risk for contracting SARS-CoV-2, the virus that causes COVID-19. Understanding the prevalence of and factors associated with SARS-CoV-2 infection among frontline HCP who care for COVID-19 patients are important for protecting both HCP and their patients. During April 3–June 19, 2020, serum specimens were collected from a convenience sample of frontline HCP who worked with COVID-19 patients at 13 geographically diverse academic medical centers in the United States, and specimens were tested for antibodies to SARS-CoV-2. Participants were asked about potential symptoms of COVID-19 experienced since February 1, 2020, previous testing for acute SARS-CoV-2 infection, and their use of personal protective equipment (PPE) in the past week. Among 3,248 participants, 194 (6.0%) had positive test results for SARS-CoV-2 antibodies. Seroprevalence by hospital ranged from 0.8% to 31.2% (median = 3.6%). Among the 194 seropositive participants, 56 (29%) reported no symptoms since February 1, 2020, 86 (44%) did not believe that they previously had COVID-19, and 133 (69%) did not report a previous COVID-19 diagnosis. Seroprevalence was lower among personnel who reported always wearing a face covering (defined in this study as a surgical mask, N95 respirator, or powered air purifying respirator [PAPR]) while caring for patients (5.6%), compared with that among those who did not (9.0%) (p = 0.012). Consistent with persons in the general population with SARS-CoV-2 infection, many frontline HCP with SARS-CoV-2 infection might be asymptomatic or minimally symptomatic during infection, and infection might be unrecognized. Enhanced screening, including frequent testing of frontline HCP, and universal use of face coverings in hospitals are two strategies that could reduce SARS-CoV-2 transmission.

HCP who care for patients with COVID-19 are at risk for exposure and infection during patient care–related activities (*1*,*2*), and once infected, can spread SARS-CoV-2 to patients, coworkers, and others in the community. Therefore, understanding the frequency of SARS-CoV-2 infection among frontline HCP and characteristics associated with infection among HCP is important for planning effective strategies for minimizing SARS-CoV-2 spread in health care settings and associated communities (*3*,*4*).

Most persons who are infected with SARS-CoV-2 develop antibodies to SARS-CoV-2 proteins within 1–2 weeks of infection (*5*). Serologic testing for SARS-CoV-2 antibodies, albeit having variable sensitivity and specificity (*6*), might provide a useful marker for identifying past SARS-CoV-2 infection. In this study, SARS-CoV-2 antibodies were measured among HCP who regularly cared for patients with COVID-19, with the aim of identifying past infection and describing characteristics associated with seropositive test results.

This study was conducted by the Influenza Vaccine Effectiveness in the Critically Ill (IVY) Network, which is a collaboration of academic medical centers in the United States conducting epidemiologic studies on influenza and COVID-19 (*1*). Thirteen IVY Network medical centers from 12 states participated.* Each hospital enrolled a convenience sample of HCP (*1*) who regularly had direct patient contact in hospital-based units caring for adult COVID-19 patients since February 1, 2020, including emergency departments (EDs), intensive care units (ICUs), and hospital wards. Targeted enrollment was 250 participants per hospital, and volunteers were enrolled during April 3–June 19. HCP who were not working because of illness or quarantine were not enrolled. Participants underwent phlebotomy for serum collection and answered survey questions about demographic characteristics, medical history, symptoms, previous clinical testing for acute SARS-CoV-2 infection, and PPE practices while caring for patients in areas with COVID-19 patients. Participants were classified as having symptoms of an acute viral illness if they reported any of the following signs or symptoms from February 1, 2020, until the enrollment date: fever (temperature >99.5°F [37.5°C]), cough, shortness of breath, myalgias, sore throat, vomiting, diarrhea, or change in sense of taste or smell. Participants were asked whether they thought that they previously had COVID-19 (*7*). Participants also self-reported PPE use in the past week and whether they personally experienced at least one episode of PPE shortage since February 1, 2020, defined as inability to access at least one of the following forms of PPE when it was wanted for patient care: surgical masks, N95 respirators, PAPRs, gowns, gloves, or face shields.

CDC received serum specimens and completed testing for SARS-CoV-2 antibodies with an enzyme-linked immunosorbent assay against the extracellular domain of the SARS-CoV-2 spike protein.^†^ This assay uses anti-pan–immunoglobulin (Ig) secondary antibodies that detect any SARS-CoV-2 immunoglobulin isotype, including IgM, IgG, and IgA. A specimen was considered reactive if it had a signal to threshold ratio >1.0 at a serum dilution of 1:100, correcting for background. Previous validation work with this assay demonstrated approximate sensitivity of 96% and specificity of 99%. Local area community incidence of COVID-19 was estimated from SARS-CoV-2 test results reported at hospital-area county public health departments. Local area community incidence was calculated as the total number of reported COVID-19 cases at the health departments from the beginning of the pandemic through 7 days after the first date of HCP enrollment at the participating hospital divided by county population and multiplied by 1,000 (*8*).

Participants were classified as having positive serology (i.e., SARS-CoV-2 antibodies detected at or above the threshold) or negative serology (i.e., SARS-CoV-2 antibodies below the threshold). Characteristics of the seropositive and seronegative groups were compared using Wilcoxon rank-sum tests for continuous variables and Pearson’s chi-squared tests or Fisher’s exact tests for categorical variables. Statistical analyses were conducted using Stata software (version 16; StataCorp). This activity was reviewed by the Institutional Review Boards at the participating medical centers and by CDC and was conducted consistent with applicable federal law and institutional policies.^§^

Among 3,248 enrolled HCP, 1,445 (44%) were nurses, 919 (28%) were physicians, nurse practitioners, or physician assistants, 235 (7%) were respiratory therapists, and 648 (20%) had other clinical roles; the clinical role of one HCP was unknown. The median age of participants was 36 years, and most (80%) reported no underlying medical conditions. Among participants, 1,292 (40%) reported working primarily in an ICU, 1,139 (35%) primarily in an ED, and 817 (25%) primarily in other locations. Among the 3,248 participants, 194 (6.0%) had detectable SARS-CoV-2 antibodies. Seroprevalence varied widely by medical center, ranging from 0.8% (three facilities) to 31.2%, with generally higher seroprevalence at medical centers within counties with high local area community cumulative incidence of COVID-19 (Figure).

**FIGURE:**
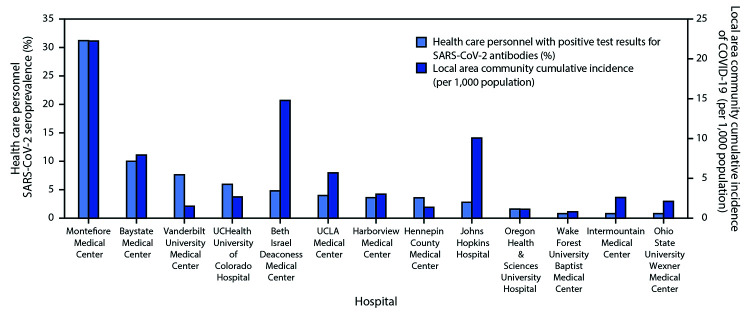
SARS-CoV-2 seroprevalence among a convenience sample of frontline health care personnel and local area community cumulative incidence of COVID-19* — 13 academic medical centers, United States, April–June 2020^†^ **Abbreviation:** COVID-19 = coronavirus disease 2019. * Calculated as the total number of reported community COVID-19 cases within a hospital-area county or counties between the beginning of the pandemic and 7 days after the first day of health care personnel enrollment at the hospital divided by population of the county or counties x 1,000. ^†^ The medical centers, counties, and dates of enrollment included: Montefiore Medical Center, Bronx, New York (Bronx, Kings, New York, Queens, and Richmond counties, May 4–5, 2020); Baystate Medical Center, Springfield, Massachusetts (Hampden County, April 22–29, 2020); Vanderbilt University Medical Center, Nashville, Tennessee (Davidson County, April 3–13, 2020); UCHealth University of Colorado Hospital, Aurora, Colorado (Adams, Arapahoe, and Denver counties, April 16–20, 2020); Beth Israel Deaconess Medical Center, Boston, Massachusetts (Suffolk County, April 20–27, 2020); UCLA Medical Center, Los Angeles, California (Los Angeles County, May 26–June 5, 2020); Harborview Medical Center, Seattle, Washington (King County, April 30–May 11, 2020); Hennepin County Medical Center, Minneapolis, Minnesota (Hennepin County, April 23–28, 2020); Johns Hopkins Hospital, Baltimore, Maryland (Baltimore County and Baltimore City, June 12–19, 2020); Oregon Health & Sciences University Hospital, Portland, Oregon (Multnomah County, May 6–7, 2020); Wake Forest University Baptist Medical Center, Winston-Salem, North Carolina (Forsyth County April 29–May 7, 2020); Intermountain Medical Center, Murray, Utah (Salt Lake County, April 30, 2020); Ohio State University Wexner Medical Center, Columbus, Ohio (Franklin, Delaware, Licking, Madison, Pickaway, and Fairfield counties, April 20–May 21, 2020).

## Characteristics of Health Care Personnel With and Without SARS-CoV-2 Antibodies

SARS-CoV-2 antibody detection differed among participants according to demographic characteristics. Seropositivity was lower among females (5.3%) than among males (7.2%) (p = 0.03) and among non-Hispanic White participants (4.4%) than among participants of other racial/ethnic groups (9.7%) (p<0.001). Symptoms of an acute viral illness since February 1, 2020, were more prevalent in participants with antibodies detected (71%) than in those without antibodies detected (43%) (p<0.001) (Table). Notably, of 194 participants with antibodies detected, 86 (44%) reported that they did not believe they previously had COVID-19, 56 (29%) reported no symptoms of an acute viral illness since February 1, 2020, and 133 (69%) had not previously had positive test results for acute SARS-CoV-2 infection. A previous positive test was reported by 61 participants, representing 31% of the 194 participants with antibodies detected and 66% of 92 participants with both antibodies detected and previous SARS-CoV-2 testing completed.

**TABLE:** Characteristics, previous symptoms, and previous testing for acute SARS-CoV-2 infection among a convenience sample of frontline health care personnel, by SARS-CoV-2 serology results — 13 academic hospitals,* United States, April–June 2020

Characteristic^†^	SARS-CoV-2 serology result, no. (%)		p-value^§^
	Positive (n = 194)	Negative (n = 3,054)	
**Median age (IQR), years**	38 (31–48)	35 (30–45)	0.077
**Sex**			
Females	113 (58)	2,014 (66)	0.029
Males	81 (42)	1,040 (34)	
**Race/Ethnicity**			
White, non-Hispanic	102 (54)	2,192 (73)	<0.001
Black, non-Hispanic	35 (19)	171 (6)	
Asian, non-Hispanic	25 (13)	340 (11)	
Other race, non-Hispanic	4 (2)	73 (2)	
Hispanic	23 (12)	228 (8)	
**Chronic medical conditions and substance use**			
Any comorbidity^¶^	37 (19)	607 (20)	0.790
Asthma	14 (7)	302 (10)	0.220
Diabetes mellitus	2 (1)	68 (2)	0.440
Hypertension	19 (10)	213 (7)	0.140
Autoimmune disease	2 (1)	88 (3)	0.170
Current smoker	3 (2)	125 (4)	0.085
**Primary location of clinical work**			
Emergency department	61 (31)	1,078 (35)	0.089
Intensive care unit	80 (41)	1,212 (40)	
Hospital ward	22 (11)	436 (14)	
Other	31 (16)	328 (11)	
**Clinical role**			
Nurse	73 (38)	1,372 (45)	0.002
Physician, nurse practitioner, or physician assistant	52 (27)	867 (28)	
Respiratory therapist	10 (5)	225 (7)	
Paramedic	3 (2)	53 (2)	
Other**	56 (29)	536 (18)	
**Typical no. of clinical workdays per week since February 1, 2020, median (IQR), days**	3 (3–5)	3 (3–4)	0.003
**Participant reported belief that he or she previously had COVID-19**	108 (56)	554 (18)	<0.001
**Specific signs or symptoms reported**			
Cough	78 (40)	780 (26)	<0.001
Sore throat	57 (29)	764 (25)	0.180
Myalgias	67 (35)	445 (15)	<0.001
Fever	58 (30)	367 (12)	<0.001
Shortness of breath	40 (21)	315 (10)	<0.001
Vomiting	17 (9)	77 (3)	<0.001
Diarrhea	38 (20)	292 (10)	<0.001
Dysgeusia	55 (28)	84 (3)	<0.001
Anosmia	54 (28)	77 (3)	<0.001
Cough or fever or shortness of breath	106 (55)	932 (31)	<0.001
Any of the above symptoms reported	138 (71)	1,309 (43)	<0.001
If any symptoms reported, time from symptom onset to serology specimen collection, median (IQR), days	30 (18–42)	34 (20–60)	0.005
**SARS-CoV-2 testing for acute infection completed clinically before serology testing^††^**			
Test not done	102 (53)	2,547 (83)	<0.001
Test done	92 (47)	507 (17)	
Test positive	61 (66% of 92 tested)	6 (1% of 507 tested)	
Test negative or indeterminate	31 (34% of 92 tested)	501 (99% of 507 tested)	

**Abbreviations:** COVID-19 = coronavirus disease 2019; IQR = interquartile range.

* Seropositive indicates that participants had antibody levels to SARS-CoV-2 detected above a threshold value, whereas seronegative indicates that antibody levels were below the threshold. Participants were from a convenience sample of health care personnel who reported regularly having direct patient contact since February 1, 2020, in units that cared for COVID-19 patients, from one of 13 academic medical centers (Harborview Medical Center [Washington], Oregon Health & Sciences University [Oregon], University of California Los Angeles [California], Hennepin County Medical Center [Minnesota], Vanderbilt University Medical Center [Tennessee], Ohio State University Wexner Medical Center [Ohio], Wake Forest University [North Carolina], Montefiore Medical Center [New York], Beth Israel Deaconess Medical Center [Massachusetts], Baystate Medical Center [Massachusetts], Intermountain Medical Center [Utah], UCHealth University of Colorado Hospital [Colorado], and Johns Hopkins Hospital [Maryland]).

^†^ Some participants had missing data for characteristics: age (25), race/ethnicity (55), clinical role (one), typical number of clinical workdays per week (five), whether or not they believed they previously had COVID-19 (one).

^§^ Wilcoxon rank-sum tests for continuous variables and Pearson’s chi-squared tests or Fisher’s exact tests for categorical variables.

^¶^ Participants were asked whether they had 11 chronic medical conditions, including asthma, chronic obstructive pulmonary disease, other chronic lung condition, chronic heart failure, coronary artery disease, diabetes mellitus, hypertension, autoimmune disease, active cancer, or an immunosuppressive condition, or required chronic renal replacement therapy (dialysis).

** Clinical role of the 56 participants with positive serology for SARS-CoV-2 who identified their clinical role as “other” included: patient care technician (22), radiology technician (11), occupational or physical therapist (eight), nursing leadership (five), social worker (three), public safety officer (two), behavioral health worker (one), chaplain (one), speech pathologist (one), housekeeping (one), laboratory technician (one).

**^††^** Six participants had negative test results for SARS CoV-2 antibodies and reported a positive clinical test for SARS-CoV-2 before serology testing; among these six participants, 20, 29, 31, 35, 36, and 46 days had elapsed from the clinical test and specimen collection for study serology testing.

## Personal Protective Equipment Use

Use of a face covering during all clinical encounters in the week preceding enrollment was reported by 2,904 (89%) participants. Detection of SARS-CoV-2 antibodies was less common among participants who reported using a face covering for all clinical encounters (6%) than among those who did not (9%) (p = 0.012). Shortages of any PPE equipment since February 1, 2020, were reported by 398 (12%) participants; shortages of N95 respirators (reported by 5% of participants) were those most commonly reported. In eight of the 13 medical centers, >10% of participants reported a PPE shortage. A higher percentage of participants who reported a PPE shortage had detectable SARS-CoV-2 antibodies (9%) than did those who did not report a PPE shortage (6%) (p = 0.009).

## Discussion

Among a convenience sample of HCP who routinely cared for COVID-19 patients in 13 U.S. academic medical centers from February 1, 2020, 6% had evidence of previous SARS-CoV-2 infection, with considerable variation by location that generally correlated with community cumulative incidence. Among participants who had positive test results for SARS-CoV-2 antibodies, approximately one third did not recall any symptoms consistent with an acute viral illness in the preceding months, nearly one half did not suspect that they previously had COVID-19, and approximately two thirds did not have a previous positive test result demonstrating an acute SARS-CoV-2 infection. These findings suggest that some SARS-CoV-2 infections among frontline HCP are undetected and unrecognized, possibly because of the minimally symptomatic or subclinical nature of some infections, underreporting of symptoms, or nonsystematic testing of some personnel with symptomatic infections.

This study resulted in the identification of two factors potentially associated with SARS-CoV-2 infection among HCP: PPE shortages and interacting with patients without wearing a face covering. These findings highlight the importance of maintaining PPE supplies at hospitals caring for COVID-19 patients and, assuming adequate supply, adhering to policies that encourage the use of masks for all interactions between HCP and patients. Universal masking has been associated with a significantly lower rate of infection among HCP (*9*).

The findings in this report are subject to at least four limitations. First, bias might have occurred if personnel at higher or lower risk for infection were less or more likely to volunteer to participate; for example, HCP not working because of illness or quarantine were not recruited and might have been at higher risk for SARS-CoV-2 infection. Second, seroprevalence could be underestimated if participants who were infected had not yet mounted an antibody response or if antibody titers had declined since infection (*10*). Third, information on facility-level infection prevention and control practices that could further affect exposure risk was not collected. Also, multivariable models to adjust for confounding were not performed. Finally, among seropositive HCP, exposure that led to SARS-CoV-2 infection could have occurred within the hospital setting or the community and this study could not distinguish between these potential sources of exposure. In general, seroprevalence among HCP across sites correlated with community COVID-19 incidence. SARS-CoV-2 exposures in the hospital could also have occurred between health care providers (e.g., within shared workspaces). 

Evidence of previous SARS-CoV-2 infection was detected in 6% of frontline HCP from 13 academic medical centers within the first several weeks of U.S. transmission, although prevalence varied considerably by location. A high proportion of personnel with antibodies did not suspect that they had been previously infected. The risk for transmission of SARS-CoV-2 from HCP to others within hospitals might be mitigated by adherence to recommended practices such as universal use of face coverings and suggestions to have dedicated cohorts of HCP caring for patients with COVID-19. In addition to maintaining PPE supplies and instituting universal face covering policies for HCP at work, enhanced screening, including frequent testing of frontline HCP, and universal use of face coverings in hospitals are strategies that could reduce SARS-CoV-2 transmission.

**Summary**

**What is already known about this topic?**

Little is known about the prevalence and features of SARS-CoV-2 infection among frontline U.S. health care personnel.

**What is added by this report?**

Among 3,248 personnel observed, 6% had antibody evidence of previous SARS-CoV-2 infection; 29% of personnel with SARS-CoV-2 antibodies were asymptomatic in the preceding months, and 69% had not previously received a diagnosis of SARS-CoV-2 infection. Prevalence of SARS-CoV-2 antibodies was lower among personnel who reported always wearing a face covering while caring for patients (6%), compared with those who did not (9%).

**What are the implications for public health practice?**

A high proportion of SARS-CoV-2 infections among health care personnel appear to go undetected. Universal use of face coverings and lowering clinical thresholds for testing could be important strategies for reducing hospital transmission.

## Footnotes

* Harborview Medical Center, Washington; Oregon Health & Sciences University, Oregon; University of California Los Angeles, California; Hennepin County Medical Center, Minnesota; Vanderbilt University Medical Center, Tennessee; Ohio State University Wexner Medical Center, Columbus, Ohio; Wake Forest University, North Carolina; Montefiore Medical Center, New York; Beth Israel Deaconess Medical Center, Massachusetts; Baystate Medical Center, Massachusetts; Intermountain Medical Center, Utah; UCHealth University of Colorado Hospital, Colorado; and Johns Hopkins Hospital, Maryland.

† https://www.biorxiv.org/content/10.1101/2020.04.24.057323v2.

§ 45 C.F.R. part 46.102(l)(2), 21 C.F.R. part 56; 42 U.S.C. §241(d); 5 U.S.C. §552a; 44 U.S.C. §3501 et seq.

## References

[1] Stubblefield WB, Talbot HK, Feldstein L, ; Influenza Vaccine Effectiveness in the Critically Ill (IVY) Investigators. Seroprevalence of SARS-CoV-2 among frontline healthcare personnel during the first month of caring for COVID-19 patients—Nashville, Tennessee. Clin Infect Dis 2020; Epub July 6, 2020. 10.1093/cid/ciaa936 32628750 PMC7454447

[2] Chou R, Dana T, Buckley DI, Selph S, Fu R, Totten AM. Epidemiology of and risk factors for coronavirus infection in health care workers: a living rapid review. Ann Intern Med 2020;173:120–36. 10.7326/M20-1632 32369541 PMC7240841

[3] Burrer SL, de Perio MA, Hughes MM, ; CDC COVID-19 Response Team. Characteristics of health care personnel with COVID-19—United States, February 12–April 9, 2020. MMWR Morb Mortal Wkly Rep 2020;69:477–81. 10.15585/mmwr.mm6915e6 32298247 PMC7755055

[4] US Department of Homeland Security. Advisory memorandum on identification of essential critical infrastructure workers during COVID-19 response. Washington, DC: US Department of Homeland Security; 2020. https://www.cisa.gov/sites/default/files/publications/Version_3.0_CISA_Guidance_on_Essential_Critical_Infrastructure_Workers_1.pdf

[5] Xiang F, Wang X, He X, Antibody detection and dynamic characteristics in patients with COVID-19. Clin Infect Dis 2020. Epub Apr 19, 2020. . 10.1093/cid/ciaa461 32306047 PMC7188146

[6] CDC. Interim guidance for COVID-19 antibody testing. Atlanta, GA: US Department of Health and Human Services, CDC; 2020. https://www.cdc.gov/coronavirus/2019-ncov/lab/resources/antibody-tests-guidelines.html

[7] Behrens GMN, Cossmann A, Stankov MV, Perceived versus proven SARS-CoV-2-specific immune responses in health-care professionals. Infection 2020;48:631–4. 10.1007/s15010-020-01461-0 32524515 PMC7286418

[8] USAFacts. Coronavirus locations: COVID-19 map by county and state. Seattle, WA: USAFacts; 2020. https://usafacts.org/visualizations/coronavirus-covid-19-spread-map/

[9] Wang X, Ferro EG, Zhou G, Hashimoto D, Bhatt DL. Association between universal masking in a health care system and SARS-CoV-2 positivity among health care workers. JAMA. 2020;324:703–704. 10.1038/s41591-020-0965-6 32663246 PMC7362190

[10] Long QX, Tang XJ, Shi QL, Clinical and immunological assessment of asymptomatic SARS-CoV-2 infections. Nat Med 2020;26:1200–4. 10.1038/s41591-020-0965-6 32555424

